# Association of prognostic nutritional index with prognostic outcomes in patients with glioma: a meta-analysis and systematic review

**DOI:** 10.3389/fonc.2023.1188292

**Published:** 2023-07-24

**Authors:** Kuo-Chuan Hung, Cheuk-Kwan Sun, Yang-Pei Chang, Jheng-Yan Wu, Po-Yu Huang, Ting-Hui Liu, Chien-Hung Lin, Wan-Jung Cheng, I-Wen Chen

**Affiliations:** ^1^ School of Medicine, College of Medicine, National Sun Yat-sen University, Kaohsiung, Taiwan; ^2^ Department of Anesthesiology, Chi Mei Medical Center, Tainan, Taiwan; ^3^ Department of Emergency Medicine, E-Da Dachang Hospital, I-Shou University, Kaohsiung, Taiwan; ^4^ School of Medicine for International, College of Medicine, I-Shou University, Kaohsiung, Taiwan; ^5^ Department of Neurology, Kaohsiung Municipal Ta-Tung Hospital, Kaohsiung Medical University, Kaohsiung, Taiwan; ^6^ Department of Neurology, Kaohsiung Medical University Hospital, Kaohsiung Medical University, Kaohsiung, Taiwan; ^7^ Department of Nutrition, Chi Mei Medical Center, Tainan, Taiwan; ^8^ Department of Internal Medicine, Chi Mei Medical Center, Tainan, Taiwan; ^9^ Department of General Internal Medicine, Chi Mei Medical Center, Tainan, Taiwan; ^10^ Department of Anesthesiology, Chi Mei Medical Center, Liouying, Tainan, Taiwan

**Keywords:** glioma, prognostic nutritional index, overall survival, progression-free survival, prognosis, nutrition

## Abstract

**Background:**

The potential link between Prognostic Nutritional Index (PNI) and prognosis in patients with glioma remains uncertain. This meta-analysis was conducted to assess the clinical value of PNI in glioma patients by integrating all available evidence to enhance statistical power.

**Method:**

A systematic search of databases including Medline, EMBASE, Google Scholar, and Cochrane Library was conducted from inception to January 8, 2023 to retrieve all pertinent peer-reviewed articles. The primary outcome of the study was to examine the association between a high PNI value and overall survival, while secondary outcome included the relationship between a high PNI and progression-free survival.

**Results:**

In this meta-analysis, we included 13 retrospective studies published from 2016 to 2022, which analyzed a total of 2,712 patients. Across all studies, surgery was the primary treatment modality, with or without chemotherapy and radiotherapy as adjunct therapies. A high PNI was linked to improved overall survival (Hazard Ratio (HR) = 0.61, 95% CI: 0.52 to 0.72, *p* < 0.00001, I^2 = ^25%), and this finding remained consistent even after conducting sensitivity analysis. Subgroup analyses based on ethnicity (Asian vs. non-Asian), sample size (<200 vs. >200), and source of hazard ratio (univariate vs. multivariate) yielded consistent outcomes. Furthermore, patients with a high PNI had better progression-free survival than those with a low PNI (HR=0.71, 95% CI: 0.58 to 0.88, *p*=0.001, I^2 = ^0%).

**Conclusion:**

Our meta-analysis suggested that a high PNI was associated with better overall survival and progression-free survival in patients with glioma. These findings may have important implications in the treatment of patients with glioma. Additional studies on a larger scale are necessary to investigate if integrating the index into the treatment protocol leads to improved clinical outcomes in individuals with glioma.

**Systematic review registration:**

[https://www.crd.york.ac.uk/prospero/], identifier [CRD42023389951].

## 1 Introduction

Gliomas, the most frequent form of primary brain tumors, are tumors of the central nervous system that originate from glial cells (1). The yearly occurrence rate is 5.26 per 100,000 individuals in the United States, resulting in approximately 17,000 fresh diagnoses annually (2, 3). Gliomas are classified into four grades (I-IV) based on their histological features and degree of malignancy with grade IV (glioblastoma multiforme, GBM) being the most malignant (4). Regular treatment methods for gliomas consist of removing the tumor through surgery, administering radiotherapy, and chemotherapy (5). Despite advances in treatment, the prognosis for glioma patients can be poor, especially for those with high-grade tumors (6). The five-year survival rate for gliomas varies based on the tumor grading, with grade I tumors having a 62.3% survival rate and grade IV GBM only having a 4.6% survival rate (7, 8). Lower-grade gliomas typically have a median survival time of 6.5 to 8 years, while GBM has a shorter survival time of approximately 1.25 years (9, 10). Early identification of patients who may have an unfavorable outcome is critical for tailoring individualized treatment regimens to improve overall survival (11).

There is a growing body of evidence suggesting that preoperative malnutrition and inflammation, such as low prognostic Nutritional Index (PNI), may be linked to poor prognosis in patients with gastric cancer, lung cancer, breast cancer, and colon cancer (12–15). The PNI, which is calculated from the following formula: 10 × serum albumin (g/dL) + 0.005 × total lymphocyte count (per mm^3^), has also been examined as a potential prognostic predictor for patients with glioma, but there have been conflicting conclusions regarding the association between PNI and prognosis of glioma patients (16–20). Although some studies have proposed that PNI is an independent prognostic factor in glioma patients (19, 20), other research has not observed any notable correlation between these markers and survival outcomes in individuals with gliomas (16–18). In order to establish their correlation, a prior meta-analysis of three cohort studies indicated that elevated PNI levels are linked to improved overall survival (21). However, the evidence may not be conclusive due to the limited number of studies. A subsequent meta-analysis published in 2020 using combined data from seven cohort studies revealed no significant association between PNI and glioma-related prognosis (22). Recently, there have been multiple cohort studies reporting on the predictive potential of PNI in relation to the prognosis of individuals with glioma (17–19, 23–25). To enhance the statistical power by integrating all available evidence, the purpose of the current meta-analysis was to assess the potential clinical value of PNI in glioma patients through a systematic approach.

## 2 Methods

### 2.1 Protocol

The methods and results of this meta-analysis were reported according to the Preferred Reporting Items for Systematic Review and Meta-analysis (PRISMA) guidelines. The current study’s protocol had been previously registered in PROSPERO (register number: CRD42023389951)

### 2.2 Search strategy

All relevant peer-reviewed articles were retrieved through searching the following databases including Medline, EMBASE, Google Scholar, and Cochrane Library from inception to January 8, 2023. We adopted the following search terms for comprehensive search: (“Glioblastoma” or “gliomas” or “Glial Cell Tumors” or “glioblastoma multiforme” or “Astrocytoma”) AND (“Prognostic nutritional index” or “Prognostic Nutritional Indices” or “PNI”) AND (“survival” or “progression free survival “ or “mortality”). There was no restriction on the language or publication date. Meanwhile, a manual screening was also conducted to identify additional articles listed in the references of relevant articles and reviews. **Supplemental Table 1** summarized the search strategies for the Medline database.

### 2.3 Criteria for inclusion and exclusion

The following inclusion criteria were used for observational studies in the analysis: (a) patients with glioma were included regardless of its grade and type of treatment; (b) baseline PNI data were available prior to the beginning of follow-up; (c) prognostic outcomes, including overall survival or progression-free survival rate, were available during follow-up; (d) univariate or multivariate hazard ratios (HRs) with 95% confidence intervals (CIs) were reported; (e) the full text of the article could be retrieved.

Studies meeting any one of the following criteria were excluded: (a) Studies with overlapping data; (b) Studies focusing on postoperative complications without the assessment of outcomes of interest (e.g., overall survival); (c) Studies published as abstracts, case series, letters, and reviews; (d) No relevant data for calculating the risk (e.g., HRs and 95% CIs).

### 2.4 Outcomes and data extraction

The primary objective of this study was to investigate the association between high PNI values and overall survival in glioma patients. Secondary outcomes included the relationship between high PNI values and progression-free survival. Two independent reviewers gathered the following information: name of the first author and year of publication, patient characteristics (age, gender), PNI cut-off value, follow-up duration, number of cases, cancer type, and the country where the study was conducted. Multivariate HRs and 95% CIs were extracted from each study. In case of unavailability, we collected univariate HRs with 95% CIs for the analysis. We attempted to contact the corresponding authors of eligible studies up to three times to obtain missing information *via* email.

### 2.5 Risk of bias assessment

The quality of each study was investigated by two independent reviewers based on the Newcastle-Ottawa Scale (NOS) criteria, which is a tool that has been verified to assess the quality of non-randomized trials. It determines the quality of the study based on three domains, which are the selection, comparability, and exposure assessment. The maximum score for the selection parameter is 4, while comparability and exposure assessment are assigned scores of 2 and 3, respectively. The total score can reach a maximum of 9, and studies that score 7 or more are of high quality. In case of any discrepancies in the quality assessment, another investigator will be consulted.

### 2.6 Statistical analysis

The current meta-analysis utilized a random-effects model to determine the association between a high PNI and prognostic outcomes. Pooled HR and 95% CI were calculated with a HR < 1 indicating a favorable prognosis associated with a high PNI. Significant heterogeneity among studies was identified if I^2^ was over 50%. Sensitivity analyses using a leave-one-out approach were conducted to evaluate the robustness of evidence, and subgroup analyses were performed based on ethnicity (Asian ethnicity and those of non-Asian ethnicity), sample size (<200 or >200), and source of HR (i.e., univariate or multivariate). Publication bias was assessed by analyzing the symmetry of the funnel plot. Statistical analysis was performed using Review Manager (RevMan) 5.3 and comprehensive Meta-Analysis (CMA) V3 software (Biostat, Englewood, NJ, USA).

## 3 Results

### 3.1 Study selection

**Figure 1:**
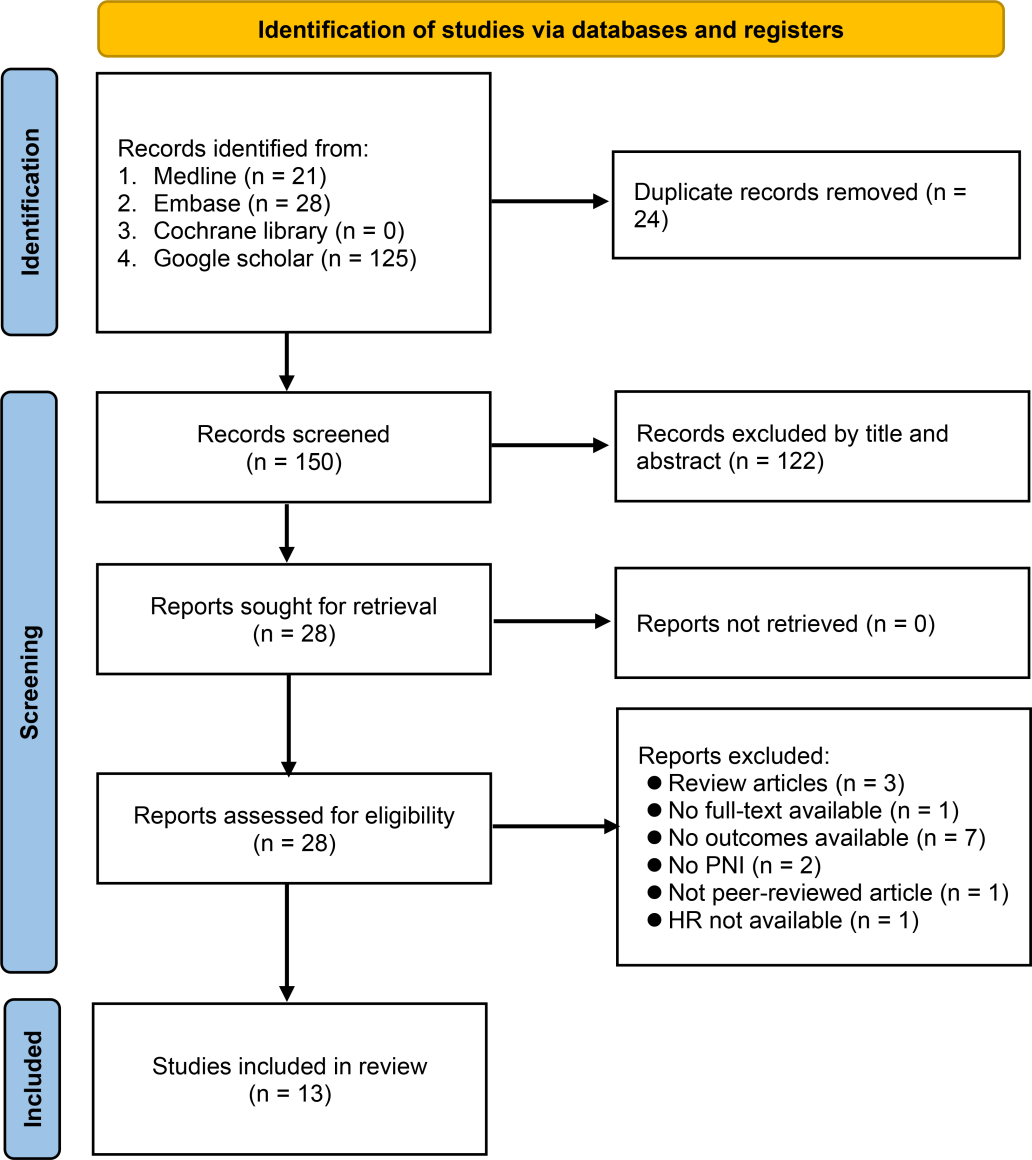
Flow chart for study selection. HR, hazard ratio; PNI, prognostic nutritional index.

Our database search retrieved 174 records, of which 24 were duplicate records. After screening the titles and abstracts of the remaining 150 records, 28 articles were extracted for full-text review. Of these, 15 citations were excluded for various reasons (**Figure 1**), leaving 13 retrospective studies published between 2016 and 2022 for inclusion in our meta-analysis (16–20, 23–30). The study selection process is summarized in **Figure 1**.

The characteristics of the included studies are shown in **Table 1**. A total of 2,712 patients were included in the analysis, with the number of patients in each study ranging from 64 to 706 (<200 in eight studies; >200 in five studies). In all studies, surgical removal of tumors was the primary treatment modality, with or without chemotherapy and radiotherapy adjuncts (**Supplemental Table 2**). The PNI cut-off values were reported in 12 studies (range: 43.38 to 52.55) (16–20, 23–26, 28–30), while relevant data was unavailable in one study (27). Information on overall survival and progression-free survival was available in 13 and four studies, respectively. Only six studies reported follow-up time (17–19, 24, 26, 28), while this information was unavailable in the other seven studies. Multivariate HR and univariate HR were provided in ten (16, 18–20, 24–26, 28–30) and three (17, 23, 27) studies, respectively. Seven studies were conducted in China (16, 20, 24, 27–30), while the other studies were conducted in Turkey (n=2) (18, 19), Italy (n=2) (17, 26), Australia (n=1) (23), and the United States (n=1) (25). The quality of studies assessed using NOS is revealed in **Table 1**, with 12 of the 13 studies considered at low risk of bias (NOS score range: 8-9) (16–18, 20, 23–30), and one study deemed to be of poor quality (NOS score: 6) (19).

**Table 1:** Characteristics of studies (n=13).

Study (Author/year)	Age (year)‡	Male	No.	PNI Cut-off value	Outcomes	Follow-up	Cancer type	HR source	Country	NOS
Alan 2022	57.5 (29–77) vs. 51 (28–74)	70.3%	64	45.7	OS	9 m	Glioblastoma (high-grade glioma)	M	Turkey	6
Ding 2018	49.9 ± 14.0	60.7%	300	44	OS	NA	GBM (IV)	M	China	8
Garrett 2021	63 (51-73)	62.1%	87	48.5	OS, PFS	NA	Grade IV glioma	U	Australia	8
He 2017	NA	NA	318	52.55	OS, PFS	NA	Gliomas (II-IV)	M	China	8
He 2021	50 (18-79)	52.7%	91	44	OS	21 m	gliomas (III-IV)	M	China	8
Huq 2021	57.6 ± 12.9	64.5%	242	43.38	OS	NA	Glioblastoma	M	USA	9
Marini 2020	<60 years: 42; ≥60 years: 82	52.4%	124	44.4	OS, PFS	1 - 4 y	GBM (IV)	U	Italy	8
Rigamonti 2019	66.4 (28.5-83.3)	63.1%	282	45.9	OS	3.3 y	GBM (IV)	M	Italy	8
Xu 2017	50.4 ± 14.5	50.6%	166	48	OS	14 m	GBM (IV)	M	China	9
Yang 2019	47.8 ± 14.0	55.5%	128	45	OS	NA	Gliomas (III-IV)	M	China	9
Yılmaz 2021	60 (20-81)	60.0%	120	46.5	OS, PFS	17 (1-67) m	GBM (IV)	M	Turkey	8
Wang 2018	45.2 ± 13.4	57.6%	706	NA	OS	NA	All gliomas (II-IV) GBM (IV)	U	China	8
Zhou 2016	53 (43-62)	59.5%	84	44.4	OS	NA	GBM (IV)	M	China	8

PFS, progression free survival; OS, overall survival; ‡mean ± sd, median (first quartile to third quartile), or number; HR, hazard ratio; NA, not available; U, univariate; M, multi-variate.

### 3.2 Outcomes

#### 3.2.1 Primary outcome: association of PNI with overall survival

**Figure 2:**
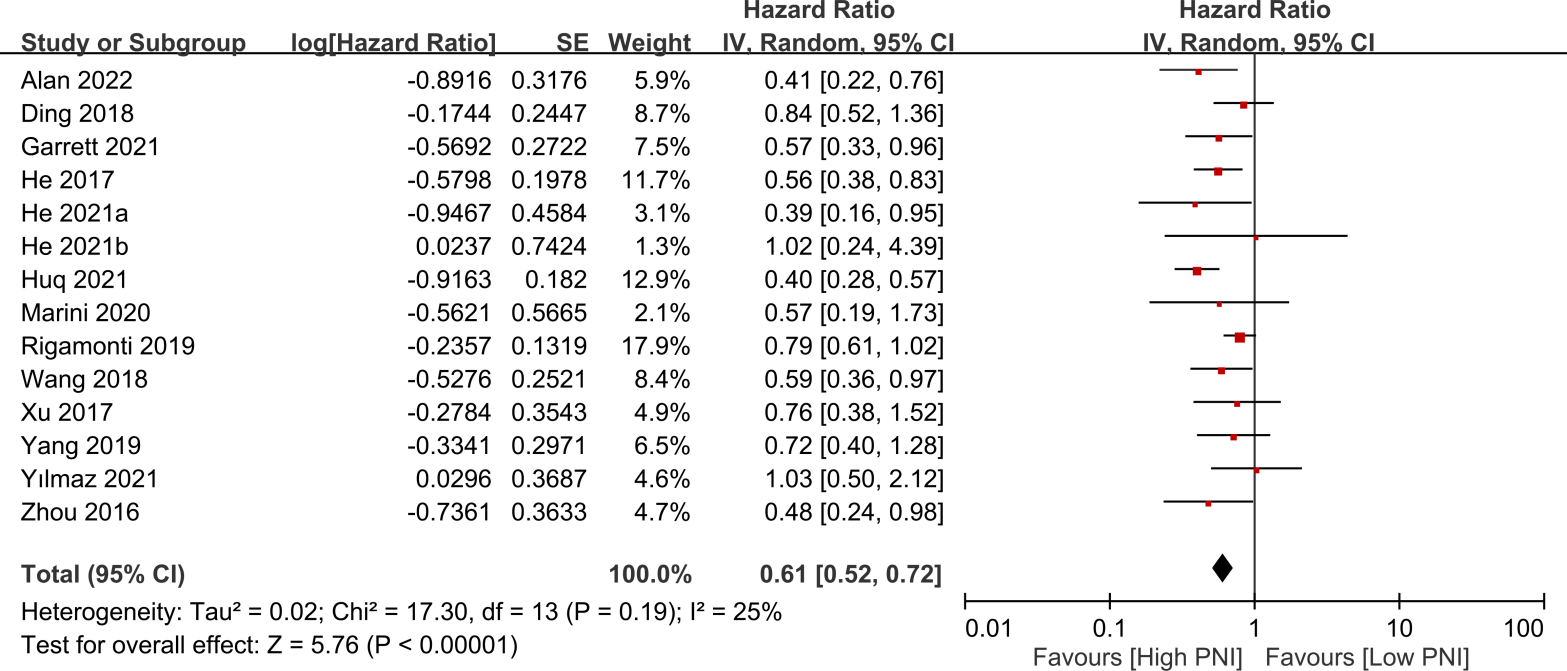
Forest plot showing a correlation between a high prognostic nutritional index (PNI) and favorable overall survival. CI, confidence interval; SE, standard error.

**Figure 3:**
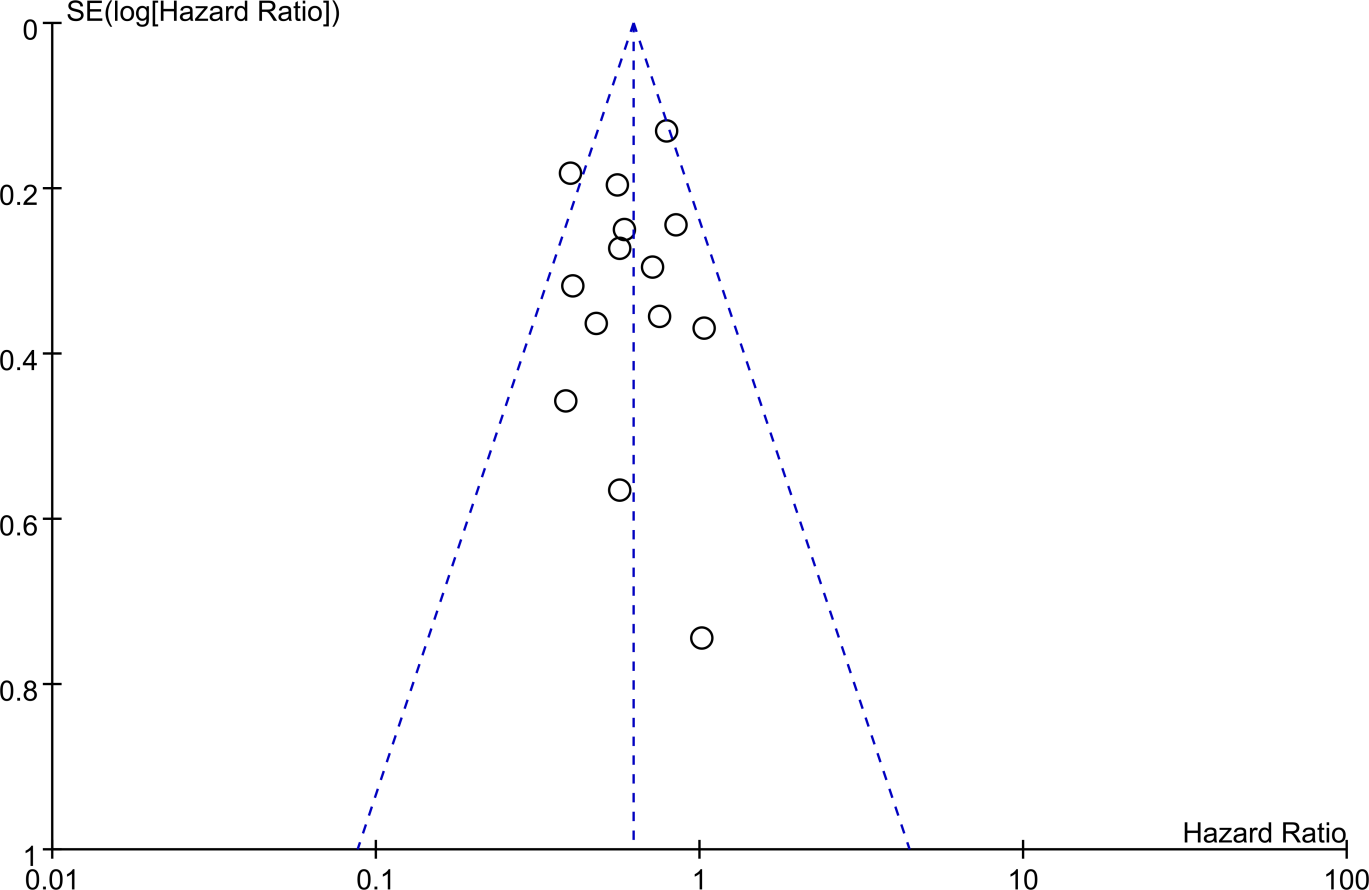
Funnel plot revealing a low risk of publication bias.

**Figure 4:**
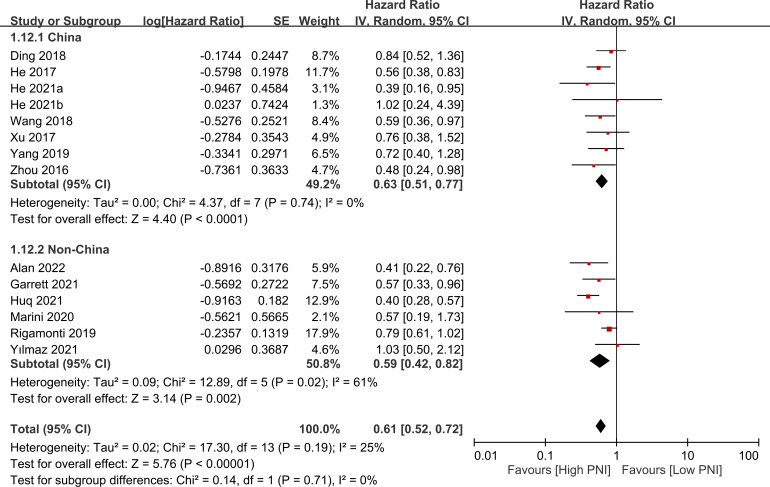
Subgroup analysis indicated that there is a positive association between a high prognostic nutritional index (PNI) and favorable overall survival, regardless of the ethnicity. CI, confidence interval; SE, standard error.

**Figure 5:**
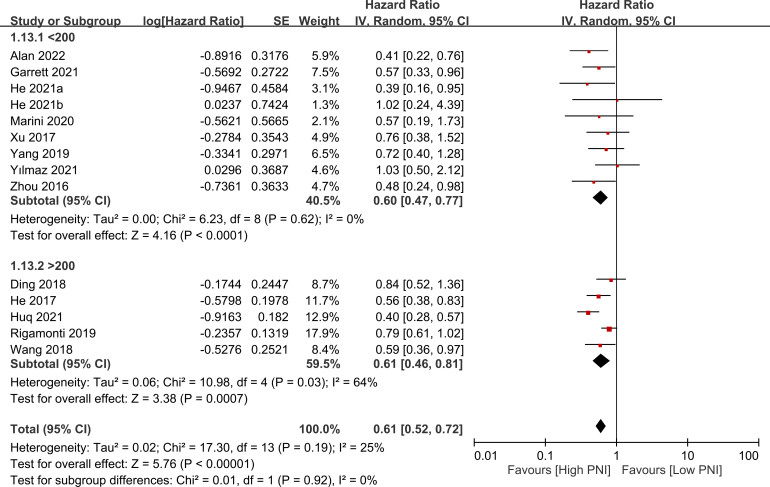
Subgroup analysis based on sample size (i.e., <200 vs. >200) showing a consistent relationship between a high prognostic nutritional index (PNI) and favorable overall survival. CI, confidence interval; SE, standard error.

**Figure 6:**
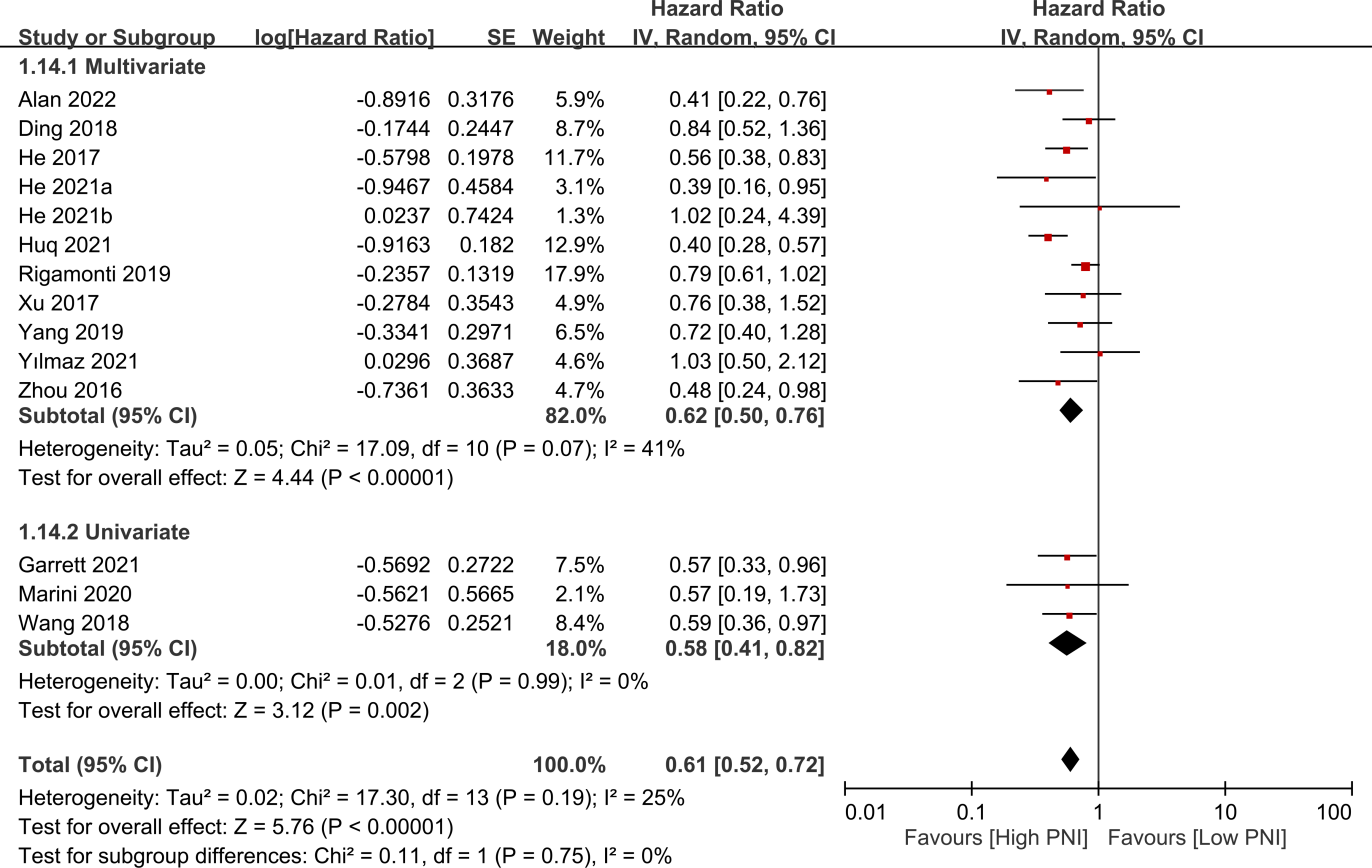
Subgroup analysis based on source of hazard ratio (i.e., univariate vs. multivariate) showing a consistent relationship between a high prognostic nutritional index (PNI) and favorable overall survival. CI, confidence interval; SE, standard error.

The association between PNI and overall survival was reported by all studies, with one study (24) providing two separate data sets that were labeled as He 2021a and He 2022b. A meta-analysis of the pooled data demonstrated that a high PNI was associated with a favorable overall survival (HR=0.61, 95% CI: 0.52 to 0.72, *p*<0.00001, I^2 = ^25%) (**Figure 2**) (16–20, 23–30). The sensitivity analysis using the leave-one-out method showed consistent results. The funnel plot indicated a low risk of publication bias in this outcome (**Figure 3**). Subgroup analyses based on ethnicity (Asian ethnicity and those of non-Asian ethnicity) (**Figure 4**), sample size (<200 vs. >200) (**Figure 5**), and source of HR (univariate vs. multivariate) (**Figure 6**) revealed a consistent relationship between a high PNI and favorable overall survival.

#### 3.2.2 Secondary outcome: association of PNI with progression free survival

**Figure 7:**
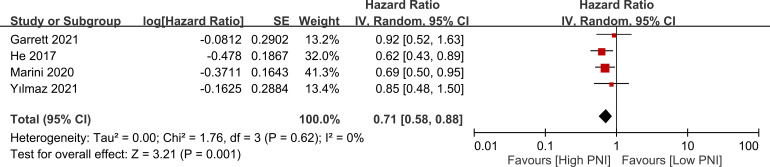
Forest plot showing a correlation between a high prognostic nutritional index (PNI) and favorable progression free survival. CI, confidence interval. SE, standard error.

Four studies presented data on the relationship between PNI and progression-free survival. High PNI was associated with favorable progression-free survival compared to low PNI (HR=0.71, 95% CI: 0.58 to 0.88, *p*=0.001, I^2 = ^0%) as shown in **Figure 7** (17, 18, 20, 23). This finding was consistent in sensitivity analysis.

#### 3.2.3 Baseline characteristics in patients with low and high prognostic nutritional index

**Supplemental Table 3** summarizes the baseline characteristics of patients with low and high PNI. There were no differences observed in gender, age, or tumor location between patients with low and high PNI. However, patients with high PNI received chemotherapy and gross total resection more frequently than those with low PNI.

## 4 Discussion

In our meta-analysis of 13 retrospective studies published between 2016 and 2022, we investigated the correlation between baseline PNI and the overall survival and progression-free survival of patients with glioma. The results of our meta-analysis showed a significant association between a high PNI and favorable overall survival. Subgroup analyses based on various factors, such as ethnicity, sample size, and source of HR (i.e., univariate vs. multivariate), consistently supported this relationship. Additionally, our analysis revealed that patients with high PNI had a more favorable progression-free survival compared to those with low PNI. Overall, our findings suggest that PNI could serve as a valuable prognostic predictor for patients with glioma, and may be considered as an important factor in clinical decision making.

Accurate prediction of glioma prognosis is critical for guiding treatment decisions (e.g., surgical planning and adjuvant treatment selection), counseling patients and their families, and optimizing healthcare resource allocation (5, 31, 32). Several predictors of prognosis for glioma have been proposed. Tumor characteristics, such as the grade of the glioma, its location within the brain, and certain biomarker genes, are important predictors of prognosis (33–36). Perioperative Karnofsky Performance Status (KPS) is also identified as a good prognosis indicator for glioma patients (33, 37). Additionally, a patient’s age, extent of surgery, functional status, and response to treatment (e.g., immediate response to radiation therapy) can all predict their likelihood of survival (33, 36, 38). These conventional predictors may provide valuable information that can help guide treatment decisions and improve patient outcomes.

Inflammation-based prognostic marker, such as the neutrophil-to-lymphocyte ratio (NLR), lymphocyte/monocyte ratio (LMR), and platelet-to-lymphocyte ratio (PLR), have been shown to be useful in predicting survival outcomes in various types of cancer, including lung cancer, colorectal cancer, breast cancer, and ovarian cancer (39–42). These indices are based on the levels of different types of cells in the blood, and can reflect the body’s immune response to the cancer. Pooled evidence through systematic approach also supports the association of these hematological indices with prognosis of cancer. For example, a previous meta-analysis that included 39 studies with a total of 17,079 breast cancer patients showed that elevated NLR and PLR were associated with poor overall survival and disease-free survival for breast cancer patients, highlighting the usefulness of these biomarkers in the management of breast cancer (43). Some studies have demonstrated that these indices are better predictors of prognosis than the tumor node metastasis (TNM) staging system or other clinicopathological variables (44, 45). Accordingly, the use of these predictors may lead to more personalized and effective treatment approaches, ultimately improving patient outcomes. Nevertheless, a meta-analysis conducted earlier investigated the effectiveness of various hematological indices, including the NLR, LMR, PLR, and found that NLR was an independent predictor of the prognosis of glioma, while PLR and LMR were not (21). The results indicate that the predictive capacity of biological markers for determining the prognosis of glioma patients varies.

The results of our meta-analysis demonstrate the efficacy of a combination of nutrition and inflammation indicators for predicting the prognosis of glioma patients. The usefulness of PNI may be attributed to the interaction between malnutrition and inflammation in cancer patients. Malnutrition, which is common in cancer patients, is associated with a weakened immune system, decreased physical activity, and muscle wasting (46, 47). These conditions can contribute to the development of chronic inflammation, which can lead to the production of cytokines and other inflammatory mediators that can promote tumor growth, angiogenesis, and metastasis. Furthermore, chronic inflammation can also suppress the immune system, leading to impaired lymphocyte function and reduced lymphocyte count, enabling cancer cells to escape from immunosurveillance (48, 49). Thus, it is reasonable to use PNI, which indicates nutritional and inflammatory status, as a prognostic factor for predicting the outcome of patients with gliomas.

Our discovery regarding the link between PNI and glioma prognosis aligns with the results of a prior meta-analysis (21). Nonetheless, the previous meta-analysis had a drawback of analyzing only three cohort studies (21). Conversely, another meta-analysis examined seven cohort studies and found no correlation between PNI and prognosis in glioma patients (22). That meta-analysis had several notable limitations that could have impacted its generalizability and applicability in clinical practice (22). One such limitation was the majority of the study populations being Chinese, which raised questions about the validity of the findings for populations of different ethnicities. Moreover, significant heterogeneity was observed in that meta-analysis (22), which could have undermined the robustness of the pooled analysis. Another concern was the limited number of patients, with only 1984 patients included in that meta-analysis (22), which could have reduced the power of the analysis. We have addressed these concerns by increasing the sample size to 2712 patients, reducing heterogeneity, and conducting subgroup analysis based on ethnicity. As a result of these improvements, the current study’s findings can be more confidently applied to broader patient populations and may serve as a more reliable source of evidence in guiding clinical decision-making.

In the present meta-analysis, it is crucial to acknowledge and consider various limitations that may impact the findings. First, the number of cohort studies in the meta-analysis remained relatively small, which may limit the statistical power and lead to overestimation or underestimation of the effect size. Additionally, as the meta-analysis only included observational studies, causality cannot be established due to confounding variables, which emphasizes the need for further large-scale studies to clarify the relationship. Second, methodological flaws such as variation in follow-up time and the use of univariate analysis in some included studies may also introduce bias into the results of the meta-analysis. It is important to consider these potential sources of bias in future studies to ensure the accuracy and reliability of the results. Third, the lack of genetic alteration data may limit the ability to control for important prognostic factors, which highlights the need for more comprehensive studies that take into account a wider range of factors that may impact survival outcomes. Finally, the study did not examine differences in comorbidities such as hypertension, insulin resistance, which may also contribute to variations in PNI and should be considered in future studies.

## 5 Conclusion

Our meta-analysis of 13 retrospective studies involving 2712 patients found a significant correlation between high prognostic nutritional index and favorable overall survival and progression-free survival in glioma patients. Subgroup analyses, including ethnicity, sample size, and source of hazard ratio, consistently supported this relationship. The results suggest that PNI could serve as a useful predictive indicator in patients with glioma, potentially impacting clinical decisions.

## Data availability statement

The original contributions presented in the study are included in the article/**Supplementary Material**. Further inquiries can be directed to the corresponding author.

## Author contributions

K-CH and C-KS: conceptualization. Y-PC: methodology and software. J-YW and P-YH: validation. K-CH and T-HL: formal analysis. C-HL and W-JC: investigation. I-WC: resources. I-WC and K-CH: data curation. K-CH, and I-WC: writing—original draft preparation. K-CH and I-WC: writing—review and editing. K-CH: visualization and supervision. All authors have read and agreed to the published version of the manuscript.
