# Supplementary material for: Association of prognostic nutritional index with prognostic outcomes in patients with glioma: a meta-analysis and systematic review
Source: Front Oncol. 2023 Jul 24;13:1188292. doi: 10.3389/fonc.2023.1188292 (PMC10411533; doi:10.3389/fonc.2023.1188292)
Supplement: Supplementary file 1 [file Table_1.docx]

**Supplemental table 1.** Search strategies

| Database | Item | Key words and Search syntax |
| --- | --- | --- |
| Medline | 1 | ("Glioblastoma" or "gliomas" or "Glial Cell Tumors" or "glioblastoma multiforme" or "Astrocytoma").mp. |
|  | 2 | exp "Glioma"/ or exp "Glioblastoma"/ |
|  | 3 | ("Prognostic nutritional index" or "Prognostic Nutritional Indices" or "PNI").mp. |
|  | 4 | ("survival" or "progression free survival " or "mortality").mp. |
|  | 5 | exp "Progression-Free Survival"/ or exp "Survival"/ or exp "mortality"/ |
|  | 6 | (1 or 2) and 3 and (4 or 5) |

**Supplemental table 2.** Study population and treatment

| Study (Author/year) | Population | Treatment |
| --- | --- | --- |
| Alan 2022 | 64 patients with recurrent glioblastoma who received systemic treatment and followed between 2012 and 2018 | Surgery and targeted therapy |
| Ding 2018 | 300 GBM patients who were surgically treated at our institute from 2008 to 2017 | Surgery and chemotherapy |
| Garrett 2021 | GBM patients (n = 87, single-centre, recruited 2013–2019) were retrospectively divided into low and high groups using literature-derived cut-offs | Surgery, chemotherapy, and radiotherapy |
| He 2017 | 331 patients with newly diagnosed WHO Grade III and IV gliomas from Jan. 1st 2001 to Jul. 31th 201 | Surgery, adjuvant radiotherapy and chemotherapy |
| He 2021 | Operable high-grade glioma patients underwent tumor resection from December 2013 to December 2019 | Surgery, chemotherapy, and radiotherapy |
| Huq 2021 | Patients with glioblastoma undergoing surgery from 2007 to 2019 | Surgery, chemotherapy, and radiotherapy |
| Marini 2020 | From January 2013 to April 2019, 124 patients operated for glioblastoma were analyzed. | Surgery, chemotherapy, and radiotherapy |
| Rigamonti 2019 | 282 individuals with a newly diagnosed GBM were enrolled between 2004 and 2014, while patients from the other hospitals were enrolled from 2007 to 2014. | Surgery, radiotherapy, and other therapy |
| Xu 2017 | 243 patients with a diagnosis of GB undergoing surgery | Surgery, chemotherapy, and radiotherapy |
| Yang 2019 | 128 patients with glioma who underwent brain tumor resection between 2008 and 2012. | Surgery |
| Yılmaz 2021 | 159 patients who were followed up with the diagnosis of GBM | Surgery, adjuvant radiotherapy and chemotherapy |
| Wang 2018 | From 2008 to 2017, patients with a pathological diagnosis of glioma who underwent surgery | Surgery |
| Zhou 2016 | Patients with GBM undergoing tumor resection between January 2013and December 2014 | Surgery, chemotherapy, and radiotherapy |

**Supplemental table 3.** Baseline characteristics in patients with low and high prognostic nutritional index

| Variables | studies | Participants | effect size | Comments |
| --- | --- | --- | --- | --- |
| Male | 8 | 1053 | 0.84 [0.57, 1.23] | No difference |
| Chemotherapy | 3 | 686 | **0.65 [0.44, 0.96]** | **Patient with high PNI receiving more chemotherapy** |
| age>60 | 3 | 336 | 0.95 [0.18, 5.12] | No difference |
| Age (mean) | 5 | 615 | 2.69 [-0.34, 5.72] | No difference |
| Gross total resection | 6 | 555 | **0.49 [0.27, 0.90]** | **Patient with high PNI receiving more gross total resection** |
| Subtotal resection | 6 | 378 | **1.83 [1.19, 2.81]** | **Patient with high PNI receiving less subtotal resection** |
| Tumor location Frontal lobe | 3 | 378 | 0.98 [0.48, 1.99] | No difference |
| Tumor location Temporal lobe | 3 | 378 | 0.88 [0.33, 2.34] | No difference |
